# Technological Innovation and Consumer Trust: Understanding Safety Perceptions in Next Generation Probiotic Development

**DOI:** 10.3390/microorganisms14071479

**Published:** 2026-07-06

**Authors:** Diana Bogueva, Svetla Danova, Mükerrem Betül Yerer, Choi Siu Mei Emily

**Affiliations:** 1 Curtin University Sustainability Policy (CUSP) Institute, Curtin University, Perth 6102, Australia; 2 Stephan Angelov Institute of Microbiology, Bulgarian Academy of Sciences, 1113 Sofia, Bulgaria; stdanova@yahoo.com; 3 Faculty of Pharmacy, Department of Pharmacology, Erciyes University, Kayseri 38039, Türkiye; eczbetul@gmail.com; 4 Department of Food and Health Sciences, Technological and Higher Education Institute of Hong Kong (THEi), Hong Kong; dremilychoi@thei.edu.hk

**Keywords:** probiotics, consumer trust, functional foods, food microbiology, safety perception, precision fermentation, microencapsulation, regulatory frameworks

## Abstract

This paper examines how technological innovation in next-generation probiotics shapes consumer trust through the lens of perceived safety. Rapid advances—spanning conventional cultures (Tier 1), postbiotics (Tier 2), and engineered microbial strains (Tier 3)—are transforming functional food architectures, yet consumer trust remains a critical determinant of their successful development, application, and adoption. Drawing on interdisciplinary evidence from food microbiology, consumer perception research, and regulatory analysis, this study examines and evaluates how these distinct technological innovation tiers alter public risk dynamics. Findings indicate that processing methodologies, media framing, and the spread of misinformation significantly influence public perceptions of microbial legitimacy, while the “Animation Gap” and “Contamination Anxiety” introduce qualitatively new cognitive friction points. Furthermore, regulatory inconsistencies across jurisdictions and variability in health claim substantiation further complicate market uptake. Streamlined case-based evidence highlights physical stability, sensory performance, and explicit value metrics that determine whether technological innovations are trusted or rejected by consumers. The paper argues that bridging the gap between scientific innovation and public acceptance requires proactive communication strategies, ethical marketing practices, and participatory engagement strategies grounded in empirical integrity. In addition, digital ecosystems, including social media and algorithm-driven content exposure, play an increasingly influential role in amplifying technology neophobia, underscoring the need for robust, targeted, evidence-based public communication in the evolving landscape of probiotic and functional food innovation.

## 1. Introduction

Probiotics are defined as “live microorganisms that, when administered in adequate amounts, confer a health benefit on the host” [1,2,3]. In recent years, the field of probiotic and functional foods has undergone a rapid technological transformation. Advances in microbiome science, microbial engineering, and novel delivery systems, such as microencapsulation and precision fermentation, are reshaping how probiotic products are designed and deployed. These innovations have significantly improved the stability, viability, and functional performance of probiotic formulations, enabling targeted applications for gut health, immune support, and metabolic regulation. Next-generation probiotics (NGPs), including commensal-derived strains and engineered microbes with targeted functionalities, represent a major shift from traditional probiotic approaches toward precision microbiome interventions [4,5]. To ensure conceptual clarity throughout this manuscript, it is necessary to define three distinct key terms representing different tiers of technological intervention:

- Next-Generation Probiotics (NGPs): Refers broadly to live microorganisms that are naturally occurring but have not traditionally been utilized as commercial probiotics, often requiring specialized anaerobic processing or target-specific gastrointestinal delivery systems.
- Advanced Microbial Solutions: An umbrella term encompassing the broader technological ecosystem, including the matrices, microencapsulation techniques, and delivery vehicles designed to protect and transport these microorganisms.
- Engineered Strains: Refers specifically to microorganisms that have undergone precise genetic or metabolic optimization—such as through synthetic biology or precision fermentation—to express enhanced functional traits or survive harsh gastrointestinal environments.

These terms are utilized distinctively herein to differentiate between the organism, the systemic delivery technology, and the genetic modification status, respectively.

Human perceptions of safety are shaped by both evolved biological mechanisms and socio-cultural influences. From an evolutionary standpoint, humans developed cognitive systems for evaluating threats and opportunities, fostering deep-seated preferences for items that signal security and familiarity [6]. However, scientific progress alone does not guarantee consumer acceptance [7]. A growing body of evidence suggests that marketplace adoption is driven less by actual safety metrics and more by perceived safety—the subjective feeling of security. In many cases, perceived safety outweighs empirical risk assessments in shaping consumer responses. Consumer decisions are often guided by trust, psychological heuristics, emotional reactions, and techno-skepticism [8] rather than systematic evaluation of raw scientific data. Such discrepancies are well-documented in research on risk perception, where individuals tend to evaluate emerging technologies using intuitive judgments and affective responses instead of technical risk analyses [9,10]. As probiotic technologies become more complex and less visible to the end-user, individuals increasingly rely on cognitive shortcuts, such as perceived naturalness, familiarity, and trust in institutions, when forming judgments about potential risks.

This dynamic creates a central tension in modern food microbiology, a paradox illustrated by engineered microbial strains, nano-encapsulation systems, and precision-fermented bioactives [7]. These advanced biotechnological interventions, although grounded in rigorous scientific validation, may conflict with intuitive consumer heuristics that associate baseline “naturalness” with safety and authenticity. Consequently, their scientific sophistication can unintentionally trigger skepticism, even when supported by robust safety evidence, clinical validation, and established regulatory frameworks such as the European Food Safety Authority (EFSA) or the US Food and Drug Administration (FDA) [11].

Within this context, trust becomes a decisive factor in adoption. Consumer confidence in probiotic innovations is shaped by the transparency of production processes, the clarity of scientific communication, the coherence of regulatory signals, and broader societal confidence in food and health institutions [9].

Regulatory frameworks play an important but uneven role in shaping this trust landscape. While institutions like EFSA apply rigorous safety and health claim assessments, global regulatory inconsistencies lead to deep variations in how probiotic innovations are marketed and perceived across jurisdictions [11]. For example, differences between the European Union’s precautionary approach and more permissive frameworks in regions such as the United States or parts of Asia can result in inconsistent product positioning and conflicting consumer messaging. These structural discrepancies reduce perceived regulatory reliability and increase consumer skepticism toward novel food technologies [12].

Against this backdrop, this article examines the interplay between technological innovation and consumer trust in next-generation probiotics, with a particular focus on perceived safety. It argues that innovation alone is insufficient. Successful adoption depends on aligning scientific advances with transparent evidence-based communication, harmonized and credible regulatory oversight, and proactive trust-building practices that address consumer concerns and reduce perceived risk. In particular, bridging the gap between scientific evidence and public understanding is critical for fostering informed acceptance. The unique contribution of this review is that it brings together technological innovation, consumer trust, and regulatory governance within a single conceptual framework for next-generation probiotics. While prior work has examined probiotic technologies, consumer acceptance, and safety regulation separately, this paper explains how these dimensions interact to shape perceived safety and market uptake. By linking case studies to the proposed framework, the review also demonstrates how transparency, familiarity, scientific validation, and institutional credibility jointly influence consumer trust.

**Figure 1:**
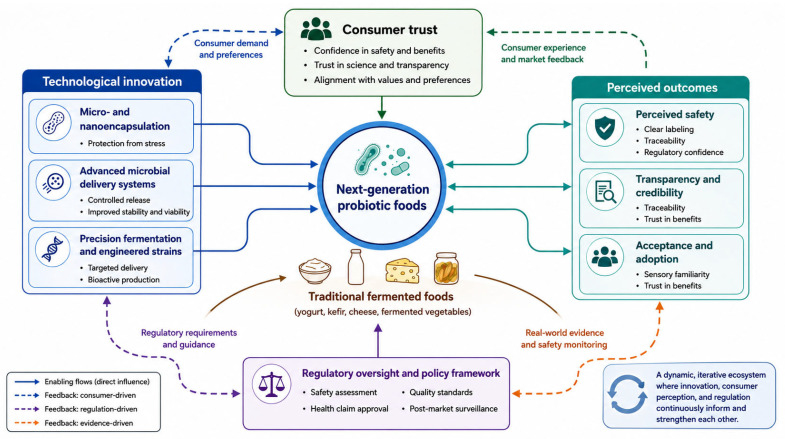
Technological Innovation and consumer trust in next generation probiotic foods.

Drawing on insights from food microbiology, consumer behavior, and regulatory science, the following sections detail how safety perceptions modulate trust and determine the market acceptance of microbial innovations, as conceptualized in Figure 1.

While prior work has examined probiotic technologies, consumer acceptance, and safety regulation separately, this paper explains how these dimensions interact to shape perceived safety and market uptake. Crucially, rather than treating probiotics as a homogeneous category, this review establishes a differentiated trust framework that separates traditional microbial cultures from next-generation interventions. We explicitly delineate how engineered strains, precision-fermented bioactives, and fastidious commensal anaerobes present qualitatively distinct trust barriers. While traditional functional foods primarily battle informational asymmetry and basic food neophobia, advanced microbial technologies trigger deeper ideological anxieties regarding naturalness, synthetic intervention, and ontological boundary-crossing—requiring fundamentally altered governance and communication architectures that transcend the basic ‘trust through transparency’ paradigm.

## 2. Technological Innovations in Next-Generation Probiotics

Technological innovation has fundamentally reshaped how probiotics are designed, produced, and delivered. The field has evolved from traditional fermentation into a highly sophisticated field that integrates microbiology, biotechnology, and food engineering. Today’s modern probiotic systems no longer focus merely on keeping bacteria alive. Instead, they focus on precision delivery and programmed functionality, transforming these microorganisms into “living medicines”, targeted functional tools [13].

Three major technological pillars define this modern landscape: micro- and nanoencapsulation, advanced microbial delivery systems, and precision fermentation. Each milestone represents a major step forward in protecting sensitive strains from processing, storage, and digestion. However, each breakthrough introduces a central paradox: as these technologies become more complex, they become less visible and intuitive to the public, creating a gap between scientific performance and perceived safety. To clearly delineate this landscape, the structural and biological transitions characterizing these platforms are mapped below.

**Figure 2:**
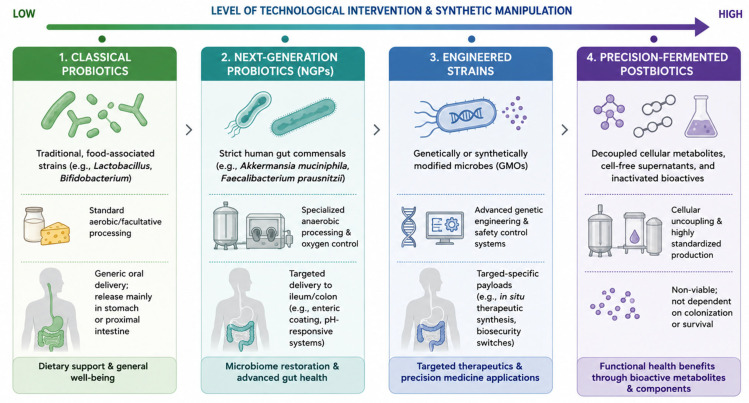
Technical taxonomy of next-generation probiotic platforms.

The schematic overview of Figure 2 illustrates the transition from classical probiotics to next-generation probiotics (NGPs), engineered strains, and precision-fermented postbiotics. As technological intervention increases, requirements for specialized processing, delivery systems, regulatory oversight, and consumer trust become more complex.

### 2.1. Micro- and Nanoencapsulation: Protecting Probiotics, Questioning Safety

Micro- and nanoencapsulation technologies are central to next-generation probiotic development, designed to protect live microorganisms from environmental stressors such as heat, oxygen exposure, moisture, stomach acidity, and bile salts, ensuring their survival through processing, storage, and gastrointestinal transit [14]. By embedding probiotic cells within protective matrices such as alginate, chitosan, lipids, proteins, and composite biopolymers, these systems enhance stability, enable controlled release kinetics, improve bioavailability, and mask undesirable sensory attributes, ultimately boosting product reliability, shelf life, and targeted delivery to the intestine [15,16,17].

From a technological standpoint, nano-scale systems, including nanoliposomes, nanoemulsions, polymeric nanoparticles, and hybrid biopolymer composites, represent a major breakthrough. They allow precise control over release, protect sensitive compounds from degradation, and increase physiological effectiveness, supporting more reliable probiotic-enriched functional foods [15,16].

However, this “engineered” sophistication introduces a critical trade-off. The further products stray from intuitively “natural” foods, the greater consumer sensitivity to processing intensity and perceived artificiality [18,19]. Nanotechnology’s unfamiliar scale amplifies risk perceptions, even with robust safety evidence, due to limited public understanding, unfamiliar terminology, and concerns over unnaturalness [20,21,22].

Practical barriers further complicate adoption, including scalability, cost, batch-to-batch consistency, maintaining probiotic viability during encapsulation, and optimizing storage conditions. Safety considerations, such as nanoparticle accumulation, long-term toxicity, unintended biological interactions, and environmental impacts, fuel both scientific debate and public skepticism, particularly for ingestible applications [23]. Regulatory authorities, including the European Food Safety Authority (EFSA) and the U.S. Food and Drug Administration (FDA), continue to refine risk assessment frameworks for nanomaterials in food applications. However, inconsistencies persist. For instance, EFSA’s 2025 guidance on the characterization of microorganisms introduces a harmonized, genomics-first risk assessment framework requiring strain-level evaluations for active agents [24,25], while the US FDA relies on distinct pre-market notifications or GRAS affirmations, causing regulatory fragmentation across different novel carrier matrices [26]. Consumers often rely on intuitive judgments and heuristic cues like perceived naturalness, familiarity, and institutional trust, making complexity a barrier to acceptance.

This trust challenge underscores that nanoencapsulation’s success hinges not just on technological refinement but on transparent risk communication, ethical marketing, and accessible explanations that translate complexity into relatable benefits, like enhanced efficacy and stability. Without these, perceived uncertainty outweighs demonstrated advantages. While nanoencapsulation offers substantial potential for controlled bioactive delivery and sensory improvements, its industrial translation remains limited, highlighting the need for complementary delivery systems that balance innovation with acceptability across diverse food matrices.

### 2.2. Advanced Delivery Systems—Enhancing Probiotic Stability and Matrix Integration

Beyond nano-encapsulation, a broad range of advanced microbial and bioactive delivery systems has emerged, including emulsions, hydrogels, liposomes, multilayer emulsions, polymeric nanoparticles, and structured biopolymer matrices. These systems act as carriers that protect probiotics and other sensitive ingredients while controlling their interaction with the host, enhancing stability, bioaccessibility, and targeted functional effects [16,27,28]. Reviews highlight how structured biopolymer matrices and multilayer emulsions can improve colonization and functional performance in next-generation probiotic formulations, enabling integration into beverages, shelf-stable products, and other non-traditional food formats [27,29,30,31].

From a food-technology perspective, these systems represent a clear step forward. Advanced delivery systems improve the functional performance of probiotics by enhancing solubility, protecting sensitive ingredients, and enabling controlled release. Emulsion-based systems, including nanoemulsions and multilayer emulsions, are particularly effective for hydrophobic compounds and probiotic-associated metabolites, while hydrogels and liposomal systems provide biocompatible and structurally flexible carriers for both hydrophilic and hydrophobic substances [32]. Biopolymer-based carriers, derived from proteins (e.g., whey and gelatin), polysaccharides (e.g., alginate, chitosan, cellulose, and pectin), and food-grade biodegradable polymers, are increasingly attractive because of their biocompatibility, sustainability, and compatibility with clean-label positioning [31,33]. Recent reviews on polysaccharide-based and biopolymer encapsulation show that composite systems can enhance encapsulation efficiency, improve thermal and acid resistance, and enable pH- or enzyme-responsive release, thereby aligning microbial delivery with the physiological environment of the gut [31,34].

Technologically, these delivery systems also support the stability of co-delivered bioactives, such as polyphenols, carotenoids, and omega-3 fatty acids, which are vulnerable to oxidation, heat, and pH-induced degradation [28]. By embedding or coating probiotics within these matrices, manufacturers can protect cells from environmental stressors (e.g., heat, oxygen, and low pH) while also improving mucosal adhesion and colonization potential [28,31]. In addition, these platforms are increasingly being explored for edible films and coatings that function both as packaging and as active delivery systems for nutrients and probiotics [35].

Advanced microbial delivery systems thus represent a powerful platform for enhancing the functional performance, stability, and targeted efficacy of probiotics in modern food systems. However, as these delivery matrices increase in architectural and chemical complexity, they fundamentally alter the baseline characteristics of the food matrix, creating a distinct socio-technical gap between laboratory efficacy and consumer alignment.

### 2.3. Precision Fermentation: When Microbes Become Designers

Precision fermentation is emerging as one of the most transformative technologies in functional food and food microbiology, with its successful implementation increasingly dependent on consumer trust, regulatory clarity, and effective public engagement. It builds upon traditional microbial fermentation processes by employing genetically engineered or optimized microorganisms, such as yeasts, bacteria, and filamentous fungi, to produce highly specific bioactive compounds, enzymes, and functional ingredients under tightly controlled conditions [36]. This approach represents a convergence of synthetic biology, metabolic engineering, and industrial microbiology, enabling unprecedented control over metabolic pathways and the production of targeted molecules at high purity, consistency, and scalability [37].

This approach allows for remarkable precision. It enables the production of bioidentical ingredients that are structurally identical to naturally occurring molecules but manufactured through microbial biosynthesis under controlled conditions, offering significant advantages in sustainability, scalability, and resource efficiency compared to conventional agricultural production systems [37]. In the context of probiotics and postbiotics, this technology is increasingly used to design microbial systems and health-relevant metabolites with targeted functional properties, including next-generation probiotic strains whose functionality can be precisely tuned rather than left to natural variation.

The process involves inserting a gene sequence encoding a desired compound into a microbial host. Once cultivated under controlled conditions, the organism expresses the compound, which is then purified for use in food products. This enables the production of dairy proteins such as casein and whey, egg-white proteins, and bioactives such as heme and omega-3 fatty acids without relying on animal agriculture. These ingredients are increasingly used in plant-based and functional formulations to improve texture, nutrition, and bioefficacy, supporting the development of allergen-friendly, cruelty-free, and climate-conscious food products [37,38].

From a technological standpoint, precision fermentation offers several compelling advantages that position it as a transformative tool in functional food development. One of its key strengths lies in the ability to produce ingredients with high purity and functionality, ensuring consistent performance across diverse food systems. These bioidentical compounds, such as proteins, enzymes, and micronutrients, can be manufactured with exceptional precision, often surpassing the variability found in conventional agricultural sources [38]. The process allows for customizable nutritional profiles and tailored production of peptides, enzymes, and other bioactives with specific health-enhancing properties, enabling the design of targeted functional foods meant to meet personalized dietary needs [39]. In terms of environmental impact, precision fermentation contributes to enhanced sustainability by significantly reducing land use, water consumption, and greenhouse gas emissions compared with traditional farming and livestock production [40]. By decoupling ingredient production from animal agriculture, it supports ethical sourcing narratives that resonate strongly with consumer values around welfare, sustainability, and allergen-conscious choices [37].

However, despite these promising benefits, consumer acceptance of precision-fermented foods remains a pivotal issue. Many consumers are receptive to sustainability and health claims, but others express caution toward foods derived from biotechnology or genetic modification, often perceiving them as “unnatural” or “synthetic”. Consumer attitudes toward precision fermentation are shaped by perceived naturalness, familiarity, and trust in food producers and regulators. A comparative study of traditional versus precision fermentation revealed that consumers often favor processes they associate with heritage and craftsmanship, while viewing newer biotechnologies with skepticism unless accompanied by transparent communication and relatable narratives [36]. Transparency in ingredient sourcing, clear labeling, and accessible education about the natural equivalence and safety of precision-fermented compounds are, therefore, essential for fostering trust and mitigating perceived distance from everyday food experiences.

A particularly important challenge for precision fermentation lies in regulatory categorization. Current evidence shows that many jurisdictions struggle to classify these ingredients, often leaving them in gray areas between Genetically Modified Organisms (GMOs), novel foods, and conventional processing aids. The lack of harmonized global regulation introduces uncertainty, delays, confusion, and risks that may further undermine consumer confidence if not clarified and addressed proactively [41]. This regulatory ambiguity amplifies communication complexity, especially when the final product is chemically identical to naturally occurring compounds, yet the production process is framed in highly technical or unfamiliar terms.

Precision fermentation thus sits at the intersection of high scientific promises and high communication sensitivity. Unlike traditional fermentation, which is culturally familiar and associated with foods like yogurt, cheese, or fermented vegetables, precision fermentation relies on engineered biological systems where microbes are effectively “programmed” to produce specific molecules. Even when the final product is safe, sustainable, and chemically identical to natural compounds, the idea of engineered microbes can create discomfort for some consumers. Research on synthetic biology and novel food technologies indicates that perceived unnaturalness and lack of transparency are major barriers to acceptance, even when sustainability and safety benefits are clearly demonstrated [21]. This means that the success of precision fermentation depends not only on the maturity of the technology itself, but also on how clearly, honestly, and empathetically it is explained to the public and embedded in trustworthy institutional and regulatory frameworks.

### 2.4. The Escalating Trust Spectrum: From Conventional Cultures to Engineered Biotherapeutics

To move beyond generalized paradigms of consumer trust, we must recognize that modern microbial innovations do not present a homogenous psychological barrier. Instead, they trigger qualitatively distinct socio-cognitive friction points based on their biological viability, genetic status, and mechanism of action. Table 1 conceptualizes this “Graduated Trust Challenge” across three distinct technology tiers.

Conventional probiotics rely on long histories of safe human consumption and established consumer familiarity. Conversely, postbiotics—defined by the International Scientific Association for Probiotics and Prebiotics (ISAPP) as “preparations of inanimate microorganisms and/or their components that confer a health benefit on the host”—fundamentally disrupt the classical “live culture” wellness paradigm. Because postbiotics achieve functional efficacy through deliberately inactivated cells or metabolites (e.g., cell-free supernatants or cellular lysates), they face an intrinsic “Animation Gap” in consumer perception. Consumers frequently struggle with the cognitive paradox of how non-replicating, “dead” material can actively support host immunity and metabolic pathways.

A distinct psychological and institutional threshold is crossed when transitioning to Engineered Live Biotherapeutics (LBPs) or “smart probiotics” developed via synthetic biology and precision metabolic engineering [42]. Unlike native strains, these organisms are rationally programmed to respond to host- or disease-associated physiological signals to synthesize targeted therapeutic cargo. This intentional genetic optimization shifts the public acceptance dynamic from localized brand transparency to complex institutional risk-governance. The consumer friction points here are characterized by deep-seated technological neophobia, synthetic modification biases, and structural anxieties regarding biocontainment, horizontal gene transfer to native gut microbiota, or environmental escape. Consequently, gaining consumer trust for engineered strains necessitates absolute reliance on rigid regulatory frameworks, such as the FDA’s clinical LBP guidelines or the EFSA’s Qualified Presumption of Safety (QPS) and Novel Food regulatory oversights, coupled with robust engineered biocontainment strategies (e.g., synthetic kill switches).

As shown in Table 1, the trust challenge shifts from a passive familiarity requirement in Tier 1 to an intellectual explanation of non-viability in Tier 2 and finally to an existential risk-benefit calculation in Tier 3. Consequently, communication strategies cannot be one-size-fits-all; they must target these highly specific cognitive friction points.

## 3. Materials and Methods

This article is a structured narrative review that synthesizes interdisciplinary literature on next-generation probiotics, consumer trust, and regulatory governance. The purpose of the review is to bring together evidence from food microbiology, food engineering, consumer perception research, and regulatory science in order to examine how technological innovation in probiotic-enriched functional foods shapes perceived safety and consumer acceptance. The review is qualitative and interpretive in nature; no original experimental data was generated.

### 3.1. Literature Search and Source Selection

To ensure methodological rigor, we adopted a structured search strategy encompassing the 2020–2025 period across major scientific databases, including Web of Science, Scopus, PubMed, and Google Scholar. We utilized a PRISMA-informed approach to filter for core interdisciplinary themes (microbiology, consumer behavior, and regulatory policy). Search terms were structured using Boolean operators (e.g., AND, OR) and included combinations of the following keywords:

(“probiotics” OR “next-generation probiotics” OR “NGPs”)
AND (“precision fermentation” OR “engineered strains” OR “microencapsulation” OR “nanoencapsulation”)
AND (“consumer trust” OR “perceived safety” OR “naturalness heuristic” OR “food technology neophobia”)
AND (“regulatory frameworks” OR “EFSA” OR “FDA” OR “risk communication”)

**Figure 3:**
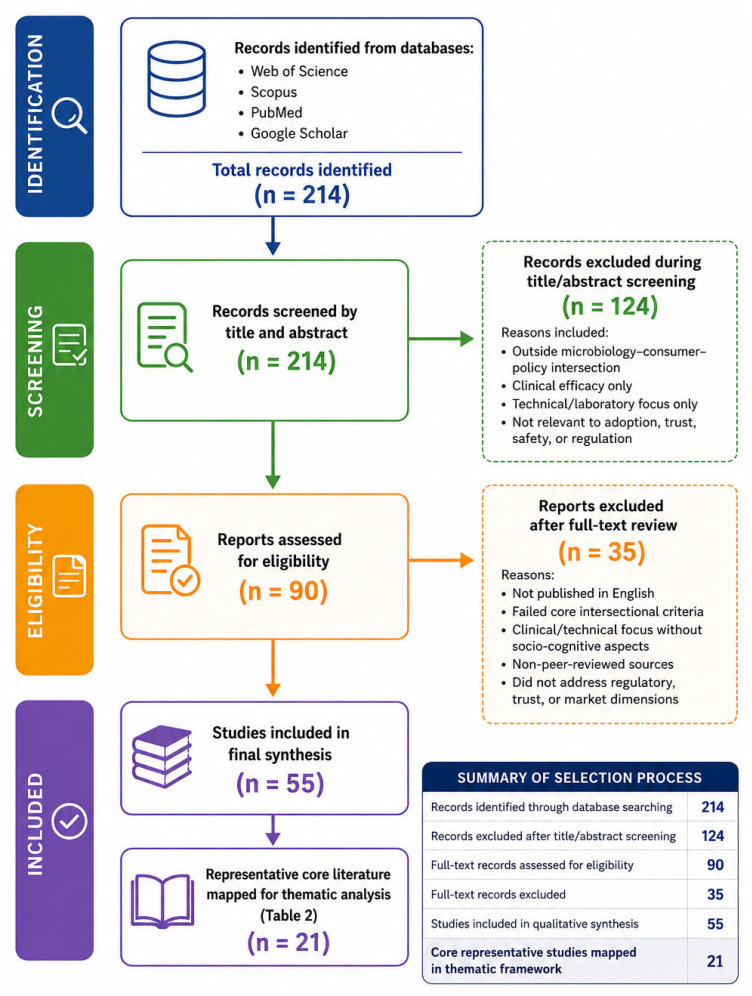
PRISMA chart.

This structured search strategy returned initial database hits, which were systematically screened based on explicit methodological boundaries. This PRISMA-informed filtering workflow is summarized in Figure 3. The stages of this process—detailed below—directly address how sources were identified, screened, and selected, and clarify the boundaries within which contradictory or divergent findings were retained rather than filtered out.

Records were first screened by title and abstract for relevance to the core intersection of microbiology, consumer behavior, and regulatory policy. Full texts were then assessed against formal inclusion criteria: sources had to be published between 2020 and 2025 as peer-reviewed journal articles, chapters, or authoritative institutional foresight reports; focus directly on next-generation probiotics, engineered strains, precision fermentation, or advanced delivery matrices; and investigate socio-cognitive barriers, sensory performance, perceived safety, trust mechanics, or regulatory governance. Sources were excluded if they were not published in English or did not address the core intersectional focus; concentrated exclusively on clinical efficacy or technical laboratory yield without socio-cognitive, trust, or market-adoption considerations; or were non-peer-reviewed, with the exception of selected white papers and institutional reports (e.g., FAO/WHO, EFSA/FDA, and the European Commission Joint Research Centre) retained for foundational regulatory baselines. This process—summarized in Figure 3—yielded a final pool of 55 primary sources, from which 21 core papers and chapters were further identified as representative target literature across the thematic categories detailed in Table 2.

### 3.2. Source Selection Rationale and Contradictory Data Handling

Peer-reviewed empirical studies, systematic reviews, and meta-analyses exploring the intersection of microbial technology and consumer behavior were prioritized. Non-peer-reviewed sources (e.g., FAO/WHO guidelines, official EFSA/FDA documentation, and industry white papers) were selectively included only when providing critical, authoritative data regarding regulatory baselines or active market parameters.

Crucially, source selection was designed to explicitly capture ideological and empirical polarization within the literature, resolving any potential selection bias. For instance, rather than omitting contradictory findings regarding consumer acceptance of precision fermentation, we deliberately balanced and juxtaposed studies showing intense technology neophobia and artificiality biases against countervailing empirical and national foresight data demonstrating an increased consumer openness to these technologies when explicitly framed around environmental sustainability and regional biosecurity benefits. Studies focusing purely on clinical efficacy without socio-cognitive or market adoption parameters were excluded.

### 3.3. Analytical Approach

The reviewed sources were organized thematically around the main topics of the article, including technological innovation, sensory performance, consumer perception, transparency and communication, and regulatory governance. The thematic synthesis followed an iterative comparative analysis technique, whereby data extracted from the 55 core sources were cross-examined to map converging consensus and diverging debates. The aim of this synthesis was to identify recurring patterns, contrasts, and conceptual linkages across literature rather than to apply rigid statistical inclusion or exclusion criteria. This approach allowed the review to integrate technical, consumer, and policy perspectives into a single conceptual framework for understanding trust in next-generation probiotic foods.

### 3.4. Data and Material Availability

All materials used in this review are publicly available and cited in the manuscript. No original datasets, laboratory materials, or custom code were created for this study. Accordingly, there are no restrictions on the availability of materials associated with this publication.

## 4. Consumer Perception and Trust in Next Generation Probiotics

Consumer trust in probiotic-enriched functional foods is shaped by a complex interplay of psychological, sensory, informational, and socio-cultural factors. As emerging technologies become more prevalent—moving from Tier 1 conventional strains to Tier 2 postbiotics and Tier 3 engineered live biotherapeutics (LBPs)—understanding how these influences interact becomes essential for fostering acceptance and mitigating skepticism. This section examines the mechanisms through which trust is strengthened or undermined, with a particular focus on how consumers evaluate the safety, naturalness, and credibility of these distinct technological tiers within increasingly technology-driven food systems.

The structural dependencies among psychological heuristics, institutional inputs, and consumer behavior are synthesized in a unified paradigm below.

**Figure 4:**
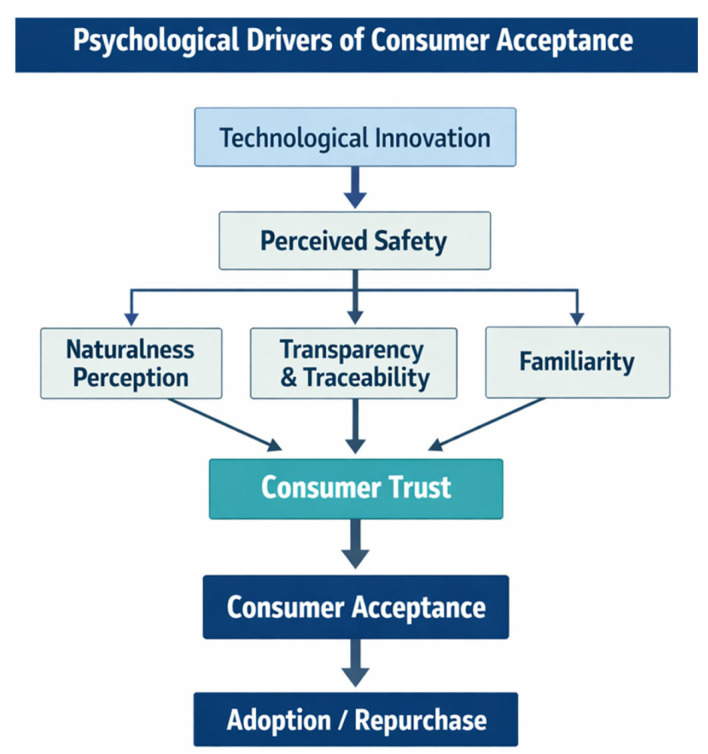
Psychological drivers of consumer acceptance.

As mapped out in this conceptual pathway (Figure 4), any baseline technological innovation must first filter through a consumer’s initial evaluation of perceived safety. This evaluation subsequently branches into three core psychological pillars that dictate public alignment: how natural the product feels, how transparent and traceable its production methods are, and how familiar the concept remains to the user’s existing dietary habits. These pillars do not operate in isolation; rather, they collectively feed into the central reservoir of consumer trust, which serves as the ultimate gatekeeper for genuine consumer acceptance and sustained, long-term market adoption or repurchase behavior. By explicitly detailing this visual hierarchy, it becomes clear that bypassing these psychological milestones can stall an innovation regardless of its scientific merit.

The qualitative shift in trust challenges becomes most apparent when contrasting the ontological status of postbiotics versus engineered strains. Postbiotics demand that consumers abandon the deeply ingrained heuristic that “live equals active benefit.” Trust here is undermined by a transparency deficit regarding processing—consumers require explicit education on how cellular lysates still interact beneficially with the host immune system. Conversely, Engineered Live Biotherapeutics (LBPs) bypass the animation gap but run directly into intense tech-neophobia. Because these organisms are genetically altered to produce specific therapeutic outcomes [42], consumer trust cannot be won merely through commercial transparency; it requires epistemic trust in regulatory gatekeepers (such as the EFSA or the FDA) to manage biocontainment and long-term ecological risks. This represents a fundamental evolution from traditional consumer brand trust to institutional risk-governance trust.

### 4.1. Health Awareness, Risk Perception, and Trust

Consumers who identify as health-oriented, those who prioritize wellness, disease prevention, and long-term physiological resilience, are particularly receptive to functional foods. Their heightened perceived vulnerability to future illness often motivates proactive, prevention-oriented dietary behaviors, such as incorporating probiotics for gut health, β-glucans for immune support, or omega-3-enriched products for cardiovascular health. This pattern aligns with findings from Baker et al. [61], who demonstrate that individuals with higher health consciousness exhibit greater willingness to adopt foods with added functional benefits. It is also consistent with broader health psychology frameworks in which perceived susceptibility and preventive motivation are key drivers of functional food uptake [62].

Importantly, this relationship is not unidirectional. Repeated exposure to probiotic and fermentation-derived products can, in turn, reinforce health awareness and deepen engagement with food-microbiological concepts. As consumers learn how microbial-derived metabolites influence gut–brain signaling or immune modulation, their sense of self-efficacy in managing health through informed dietary choices increases [63]. This bidirectional feedback loop enhances both trust and perceived control over personal health, positioning functional foods as tools for primary prevention rather than as temporary “fads.” At the same time, individuals with higher health literacy are better equipped to critically evaluate health claims, distinguish evidence-based messaging from marketing hype, and make informed dietary decisions [64]. This evaluative capacity helps embed probiotic innovations within a broader systems-oriented approach to health, integrating nutrition, microbiome modulation, and metabolic regulation [65].

From a product-development and policy perspective, these dynamics underscore the need for transparent, mechanistic explanations of how probiotic technologies work. In the absence of clear, accessible communication, consumer trust can erode even when products are scientifically validated and regulatory-compliant because the gap between what is known and what is understood widens.

### 4.2. Neophobia, Naturalness, and Technological Trust

Food neophobia—the reluctance to try unfamiliar foods—and food technology neophobia represent persistent psychological barriers to the acceptance of probiotic and functional food innovations. Even among health-motivated consumers, trust can weaken when food technologies appear overly engineered, artificial, or difficult to understand. Technologies such as nanoencapsulation, engineered probiotic strains, and precision-fermented postbiotics may evoke caution or anxiety, particularly when consumers lack familiarity with microbial and biotechnological processes. In this context, perceived naturalness emerges as a central determinant of acceptance [21,61,66,67,68]. When products are perceived as overly processed or technologically complex, perceived risk increases and willingness to consume decreases.

Empirical studies show that individuals with higher levels of food technology neophobia are less likely to accept advanced microbial formulations, especially when their understanding of the underlying mechanisms is limited [69,70,71]. Neophobia does not simply increase perceived risk; it shifts overall attitudes toward rejection even in the presence of strong scientific validation [69,71]. Crucially, this neophobic response manifests differently across the innovation tiers: Tier 2 postbiotics trigger an “Animation Gap” bias, where consumers exhibit intuitive skepticism toward the efficacy of a non-viable or “dead” microbial culture. Conversely, Tier 3 engineered strains trigger an “artificiality” bias, rooted in deep-seated resistance to synthetic biology in food production. As demonstrated by Bogueva and Danova [36] in their comparison of fermentation technologies, consumer trust drops significantly when a process crosses from traditional, nature-aligned fermentation to precision or engineered alternatives unless the underlying mechanisms are made transparent. This calls for communication strategies that translate microbial complexity into familiar conceptual frameworks, using analogies, culturally grounded stories, and visual metaphors to reduce intuitive resistance and make next-generation probiotic technologies more relatable [36,63].

### 4.3. Trust in Institutions, Science, and Information Sources

Consumer trust in probiotic innovations is closely linked to perceptions of the actors involved in their development and regulation, including scientists, food companies, and regulatory authorities. Consumers are more likely to accept novel probiotic technologies when these actors are perceived as competent, ethical, and transparent [61,69].

Key determinants of trust include traceability, transparency, and institutional credibility, all of which shape confidence in microbial food systems [21,69]. Conversely, when information is perceived as overly technical, incomplete, or opaque, consumers may infer that risks are being concealed, thereby reducing trust [67,72].

Recent research further distinguishes between cognitive trust (belief in scientific competence), affective trust (emotional reassurance), and dispositional trust (general trust in institutions and information sources) [73]. Scientific details may resonate strongly with cognitively oriented consumers, while emotionally framed messages may be more effective for those driven by affective trust. However, mismatched communication, either overly technical or overly simplified, can undermine credibility rather than strengthen it [74].

Institutional trust also plays a decisive role. When regulatory bodies are perceived as transparent and scientifically rigorous, perceived risk decreases and acceptance increases. Cross-cultural evidence supports this relationship, showing that food safety trust is also shaped by broader societal and cultural frameworks [75]. Post-pandemic trends further reinforce reliance on regulatory oversight and scientific validation, with expectations for transparent communication in food safety governance [76].

### 4.4. Familiarity, Experience, and the “Naturalness” Heuristic

Familiarity is one of the most consistent predictors of consumer acceptance in functional food systems. Probiotic delivery through familiar food matrices, such as yogurt, kefir, or fermented products, creates a psychological safety buffer that enhances perceived trust and reduces uncertainty. In contrast, unfamiliar combinations, such as nano-encapsulated probiotics in novel snack formats, may increase cognitive load and reduce acceptance. Research indicates that less processed and culturally familiar carriers are perceived as safer and more authentic [21,61].

Prior experience further reinforces acceptance. Consumers who have previously consumed probiotic products and experienced positive outcomes develop what can be described as a “trust portfolio,” increasing openness toward new formulations [21]. Repeated exposure in the absence of adverse effects reduces perceived novelty risk and transforms abstract technological novelty into normalized consumption patterns over time.

Perceived naturalness remains a dominant factor in shaping risk perception, as the naturalness heuristic functions as a cognitive shortcut where consumers equate an ‘untouched’ biological state with inherent safety [68]. This “naturalness heuristic” leads them to favor products framed as “naturally fermented” over those described as “engineered” or “lab-produced”, even when scientific evidence shows no meaningful safety difference [36].

### 4.5. Sensory Quality, Transparency, and Communication Design

Sensory attributes, including taste, texture, aroma, and appearance, remain among the strongest determinants of functional food acceptance. Even when health benefits are clearly understood, consumers tend to reject products that are perceived as unpalatable or off-putting [72]. Deviations such as overly sour, medicinal, or artificial flavors can undermine both product acceptance and trust in the brand [21,62]. This sensory hurdle is amplified by the physical delivery system used. Advanced microencapsulation or precision fermentation and nanoencapsulation architectures can profoundly alter the oral processing and mouthfeel of a food matrix, making multidisciplinary sensory design critical to consumer adherence [55]. Technical ingredient descriptions (“microencapsulated Lactobacillus strains,” “postbiotic extracts”) can increase perceived complexity and trigger suspicion if the expected sensory experience is compromised [15,28].

Expectation management is thus critical. When sensory experience aligns with marketing claims, trust is reinforced; when discrepancies occur, credibility is weakened. Probiotic yogurts, for example, demonstrate that combining indulgent taste with scientifically credible health benefits is essential for sustained market success [21,61]. Increasingly, consumers expect “no-compromise” functional foods that deliver both sensory pleasure and physiological benefit [62].

In contrast, when functional foods compromise sensory quality, consumers often perceive a trade-off between health and enjoyment, which can weaken trust in both the product and its claimed benefits [61]. Consumers frequently interpret unfamiliar sensory cues as indicators of technological manipulation, reducing acceptance of microbial innovations [77]. Familiar flavor profiles and traditional carriers (e.g., fermented dairy products) therefore play a critical role in facilitating acceptance.

Yet from a consumer perspective, advanced technical sophistication can make food feel more “engineered” and less recognizable. A yogurt containing novel multilayer emulsions, hydrogel-based carriers, or layered biopolymer-structured matrices does not evoke the same intuitive sense of safety or simplicity as a traditional matrix. This reflects the “naturalness heuristic,” where familiarity strongly shapes perceived safety and acceptability [19,20]. When technological complexity becomes invisible at the microbial level but highly complex at the formulation level, consumers rely more heavily on heuristic cues such as perceived naturalness, familiarity, trust in brands, and confidence in regulators than on technical evidence [21,63,66,78]. Trust is not built solely on scientific validity but also on whether a product feels understandable and close to everyday experience.

Ingredient transparency is also a foundational requirement for trust. Confidence increases when consumers clearly understand both what is included in the product (e.g., probiotic strains, β-glucans, fermentation-derived metabolites) and how it is produced (e.g., microencapsulation, precision fermentation, controlled microbial processing). When processing methods are concealed or communicated through opaque, technical jargon, trust tends to deteriorate [21]. Source credibility also matters. Consumers consistently place greater trust in information from academic institutions, healthcare professionals, and regulatory authorities than in marketing-driven messages [61,66]. Digital traceability tools, such as blockchain-enabled ingredient tracking, are increasingly perceived as enhancing transparency and authenticity [79].

Excessively technical language or high-information-dense ingredient lists can increase cognitive load and confusion and reduce consumer comprehension and decision-making quality, a phenomenon sometimes described as label fatigue [80]. Experimental evidence shows that increasing label complexity leads to cognitive overload and lower recall performance [81]. When consumers struggle to interpret terms such as “liposomal nano-carrier” or “precision microbial fermentation system”, they may infer that risks are being obscured, even if the technology itself is well understood scientifically. Narrative-based communication has also proven effective; using relatable metaphors such as “protective micro-bubbles surrounding probiotic cells” has proven effective in reducing cognitive distance and improving understanding [82]. Additionally, third-party validation, certifications, and references to peer-reviewed research further enhance credibility and support informed decision-making. Surveys indicate that a majority of consumers consider trustworthy food information essential in purchasing decisions [83].

Because consumer acceptance is shaped by how technology is developed and communicated, emerging research on co-design and participatory innovation suggests that involving consumers early in the development of advanced probiotic delivery systems can increase familiarity, reduce perceived risk, and strengthen trust [63,84]. When consumers are part of the design process, they are more likely to perceive new technologies as responsive to their preferences rather than imposed from the top down. Such approaches align with broader calls for “responsible innovation” governance in food and health technologies, where transparency, scientific integrity, and stakeholder engagement are integrated into R&D pathways [84,85,86].

### 4.6. Price, Value, and Socio-Cultural Context

Functional foods often command a price premium due to higher production costs associated with processes such as microbial cultivation, advanced encapsulation technologies, and regulatory compliance. However, consumer willingness to pay is driven more by perceived value than by price alone. When health benefits are clearly communicated, scientifically credible, and personally relevant, such as improvements in digestion, immunity, microbiome support, etc., consumers are more likely to accept higher prices. This economic dynamic becomes highly polarized when evaluating precision-fermented or engineered ingredients. While a premium for standard probiotics is easily accepted due to historical safety familiarity, the price premium for synthetic biological solutions can amplify skepticism if claims appear vague [87]. However, a critical divergence occurs when consumer values shift toward ethical parameters: as documented in recent food foresight frameworks [37], consumers express a significantly higher willingness to absorb premium costs for precision fermentation when the product value is explicitly tied to environmental sustainability, climate resilience, and animal-free production metrics. Conversely, when trust in the technology, ingredients, or producing company is limited, the price premium can weaken the translation of trust into actual purchase behavior.

Economic uncertainty amplifies price sensitivity. However, consumers still justify premiums when products align strongly with personal health goals or ethical values [88]. In probiotic markets, transparent communication, robust scientific validation, and consistent sensory quality are therefore essential to justify premium pricing and sustain long-term consumer trust.

Consumer trust in probiotic technologies varies across socio-demographic and cultural contexts. In societies characterized by lower levels of institutional trust, consumers tend to rely more heavily on informal networks, personal experience, and culturally embedded food practices when evaluating food-related risks and benefits [89]. Conversely, where regulatory institutions are perceived as transparent and reliable, consumers are more receptive to scientific communication and technological innovation in the food sector [69]. Empirical evidence also shows that trust in food system actors, such as regulators, producers, and certifying bodies, is a key determinant of confidence in food technologies and product integrity [90].

Beyond technological unfamiliarity, these perceptions are often linked to broader socio-ethical concerns, including fears of corporate control, reduced food sovereignty, and lack of transparency. Consumers may perceive novel food technologies as opaque or industry-driven, particularly when technological literacy is low [91]. In such contexts, affective responses, such as discomfort or uncertainty, can override analytical evaluation and scientific reasoning [92]. Gender differences in acceptance are generally modest; however, women may exhibit slightly higher levels of risk perception, particularly in relation to novel or highly processed food technologies. Overall, perceived naturalness, familiarity, and trust in institutions remain stronger and more consistent predictors of consumer acceptance than gender alone [21].

Age, education, and cultural values further shape patterns of acceptance. Younger and more educated consumers tend to be more open to probiotic and functional food innovations, particularly when these products align with health, wellness, and sustainability-related values. Research indicates that younger consumers are often more willing to try novel and innovative food products due to greater openness and curiosity toward new food technologies, while higher educational levels are consistently associated with greater acceptance and purchase intention for functional foods [61]. In addition, consumers with higher levels of nutrition knowledge and health awareness are more likely to recognize and value the benefits of probiotic products, further increasing their willingness to adopt such innovations [93]. Cultural orientation also matters—collectivist societies emphasize tradition and shared food practices, while individualistic societies foreground experimentation and innovation. These frameworks influence how probiotic technologies are interpreted and integrated into everyday diets.

### 4.7. Trust over Time—Emerging Technologies and Evolving Risk Horizons

Trust in next-generation probiotic technologies develops over time and is closely linked to perceived product maturity and accumulated safety evidence. Emerging technologies such as precision fermentation and engineered probiotics typically face a higher level of skepticism in early adoption phases, with trust gradually increasing as familiarity, real-world use, and regulatory validation accumulate [89,94].

This process reflects the interplay of epistemic trust (confidence in scientific knowledge) and social trust (confidence in institutions and supply chain actors), both of which shape perceived risks, benefits, and the acceptance of food technologies [95]. As products demonstrate long-term safety and consistent performance, perceived risk declines, and acceptance increases. Regulatory oversight and transparent communication are critical in accelerating this transition.

Early adopters play a particularly important role in shaping broader acceptance dynamics. By sharing positive experiences within social networks, they function as informal “trust amplifiers,” influencing the diffusion of probiotic innovations across consumer populations [21]. Evidence from cellular agriculture and precision fermentation shows that initial skepticism tends to diminish as real-world experience and regulatory endorsement accumulate [77]. In this way, time functions as a structural component of trust formation, reinforcing the importance of sustained transparency, long-term safety communication, and participatory engagement with consumers as technological innovation proceeds.

## 5. Transparency, Media Narratives, and Trust

Consumer perceptions of probiotic-enriched functional foods are shaped by psychological predispositions, sensory expectations, and socio-cultural values but also by the broader informational environment in which these products are framed and discussed. Trust is profoundly influenced by how probiotic innovations are communicated through labeling practices, traceability systems, and media narratives, both in traditional outlets and on digital platforms. The way microbial technologies are described, how production processes are disclosed, and how stories circulate across social channels can either reinforce confidence or deepen skepticism about the safety, authenticity, and legitimacy of next-generation probiotics [69]. This section explores how mechanisms of transparency and media dynamics interact to shape public trust in these emerging microbial technologies.

Within this context, transparency remains one of the most effective levers for building consumer trust in probiotic products. Increasingly, consumers expect more than just a list of ingredients. They seek detailed insight into production processes, including microbial origins, fermentation conditions, and safety controls [96]. For products that incorporate advanced technologies, such as microencapsulation, engineered strains, or precision-fermented compounds, traceability becomes especially important in reducing uncertainty and supporting informed decision-making [97]. Advanced traceability systems, including blockchain-enabled supply chains and QR-linked digital ingredient pathways, enable consumers to access detailed, batch-level information on sourcing, production conditions, and quality assurance protocols [98]. By transforming complex microbial processes into accessible and verifiable narratives, such systems enhance perceived authenticity and strengthen the legitimacy of novel biotechnologies.

However, transparency is effective only when it is accessible. Overly technical language and labeling can overwhelm or alienate consumers, while excessive simplification risks appearing evasive and undermining credibility. A layered communication approach is required, in which clear, concise, plain-language front-of-pack information is complemented by optional, in-depth digital content. This allows consumers to engage with information according to their preferred level of detail and complexity. This digital entry point is critical, as modern algorithmic content exposure on social media can amplify consumer anxieties or spread mischaracterized health claims. Studies evaluating probiotic-related digital media indicate substantial variability in information quality across online platforms, presenting significant challenges for ensuring the visibility of credible, evidence-based communication [99].

In probiotic contexts, transparency is most effective when paired with narrative explanation. Communicating how specific microbial strains are selected, how they are cultivated, and how they contribute to particular health outcomes helps consumers understand not only what is in the product, but also why it matters, its purpose, and its value. This “how + why” framing supports the gradual formation of confidence and aligns technical innovation with everyday food experiences and decision-making processes [96].

While transparent labeling lays a foundation for trust, social media and other digital platforms can either reinforce or destabilize that foundation. Platforms such as Instagram, TikTok, and YouTube have become central vectors for dietary health information, yet their architectures facilitate the rapid diffusion of algorithmically amplified content [100,101]. Although structural analyses of these platforms frequently rely on general digital media data, their findings directly explain the unique vulnerability of biotechnology-based functional foods to polarizing narrative frames. For example, general fear-based media frames translate directly into consumer anxieties that portray next-generation microbial innovations as unsafe or inherently “unnatural” [102]. Similarly, while macro-level studies analyze how general influencer economies drive commercial hype, this dynamic specifically manifests in the probiotic sector as exaggerated “miracle solutions” messaging that ultimately erodes long-term trust when real-world efficacy falls short [103]. Furthermore, foundational media research into conspiracy narratives underscores how public anxieties regarding opaque, corporate motives are readily mapped onto complex, invisible bioengineering processes, transforming technical uncertainty into active suspicion regarding institutional transparency [104].

These narrative frames tap into powerful emotional responses, such as fear, distrust, or fascination, and consistently outperform balanced, evidence-based scientific communication in terms of user engagement. While the structural mechanics of these engagement metrics are documented by macro-level communication studies [105], their real-world impact is uniquely acute for food biotechnology. Because consumers rely heavily on cognitive shortcuts like the “naturalness heuristic” when evaluating living, functional microbial strains, general algorithmic optimization for high-arousal content creates a non-linear feedback loop. This systemic digital architecture disproportionately scales up marginal tech-neophobic anxieties into mainstream health concerns long before institutional risk communication or formal scientific counter-narratives become visible to the public. At the same time, social media is not inherently detrimental to trust formation. When strategically used, it can become a powerful tool for transparent science communication. Educational content, for example, short-form, accessible explainer videos, can demystify complex technologies such as encapsulation or fermentation and thereby improve consumer comprehension of otherwise invisible microbial technologies [106].

**Figure 5:**
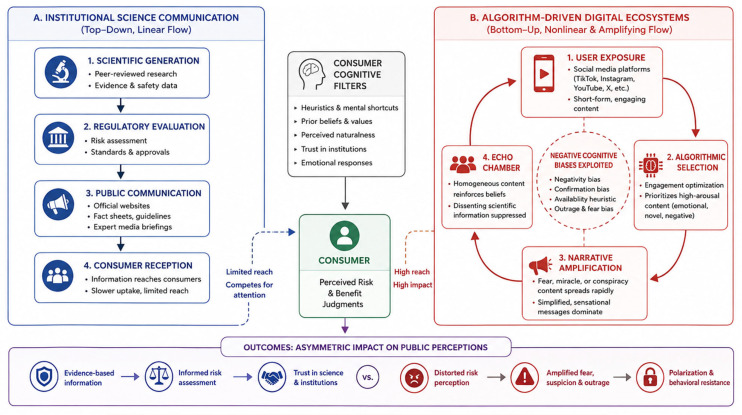
Communication vectors and algorithmic information flows.

Figure 5 illustrates how communication pathways influence consumer perceptions of next-generation probiotic technologies. Traditional institutional communication follows a linear pathway in which scientific evidence, regulatory assessment, and public communication contribute to informed decision-making. In contrast, digital ecosystems operate through non-linear, algorithm-driven feedback loops that prioritize highly engaging content. Fear-based narratives, exaggerated health claims, and conspiracy-oriented messages are amplified through echo chambers and filter bubbles, thereby increasing perceived risk and reducing trust in emerging probiotic innovations. The figure further highlights how transparency, traceability, evidence-based storytelling, expert engagement, and digital literacy initiatives can mitigate misinformation and support informed consumer acceptance.

Traceability storytelling, which follows ingredients from microbial origin to final product, enhances perceived authenticity and aligns technical innovation with consumer values [96]. Direct expert engagement by scientists, regulators, and healthcare professionals can provide accessible, evidence-based perspectives that counter misinformation without sounding dismissive or overly technical [102]. Interactive dialog formats, such as live Q&A sessions, citizen-science initiatives, and participatory communication channels, allow consumers to engage directly, ask questions, express concerns, and feel heard, thereby strengthening trust through dialog [101].

Overall, proactive narrative-driven engagement tends to be more effective than reactive myth-busting. Embedding transparency into both product design and communication strategies enables companies and institutions to leverage social media as a trust-building resource rather than simply a source of reputational risk [107]. In the context of next-generation probiotic innovations, this means aligning technical sophistication with communicative clarity, ensuring that transparency is not only visible on the label but also visible and credible in the digital ecosystems where consumers form, share, and solidify their beliefs.

Furthermore, the architecture of digital ecosystems fundamentally shapes public acceptance of food technologies through algorithmic amplification. Recommendation systems on platforms such as TikTok and Instagram prioritize high-arousal content, which structurally favor sensationalized risk narratives over nuanced scientific consensus. When users engage with content framing advanced microbial technologies as “synthetic manipulation,” algorithms reinforce exposure through feedback loops, contributing to the formation of self-reinforcing echo chambers. As documented by Bogueva and Danova [36], such patterns of digital exposure can significantly distort public perception and accelerate technology-related neophobia toward precision fermentation before formal regulatory or scientific clarifications reach mainstream audiences. Consequently, digital ecosystems function not merely as communication channels but as active socio-technical systems that shape the baseline conditions of consumer trust.

### Limitations of Transparency-Based Trust Building

Although transparency is widely regarded as a prerequisite for trust, disclosure alone does not guarantee consumer acceptance. A growing body of evidence from studies on genetically modified foods, cultured meat, and precision fermentation indicates that consumers may continue to reject novel food technologies even when comprehensive information on safety, production processes, and regulatory oversight is provided. In such cases, resistance is often rooted in underlying moral values, perceptions of naturalness, cultural identity, or broader ideological concerns rather than informational deficits.

Consequently, trust-building strategies based solely on education and disclosure may have limited effectiveness among consumers whose objections are value-based rather than knowledge-based. This suggests that transparency functions primarily as a necessary but insufficient condition for trust formation, rather than a standalone determinant of acceptance. This distinction highlights an important limitation of the transparency-centered approach proposed throughout this review and suggests that future communication strategies must extend beyond information provision to also engage with ethical, cultural, and emotional dimensions of food technology acceptance.

## 6. Case Studies of Successful and Failed Probiotic Launches

Case studies offer concrete insight into how technological innovation, communication strategies, sensory performance, and regulatory alignment shape consumer trust over time. Evaluating these market entry points demonstrates that trust within live microbial and inanimate postbiotic markets is earned through consistent quality, transparent communication, and cross-vector alignment with existing consumer heuristics.

### 6.1. Successful Case: Yakult—Trust via Simplicity and Scientific Continuity

Yakult represents an enduring model of global consumer trust sustained across eight decades by prioritizing simplicity, consistency, familiarity, and a focused scientific identity. The platform is anchored entirely on a single, well-defined strain—*Lactobacillus casei* strain *Shirota—*isolated by Dr. Minoru Shirota in 1930 [108,109]. Rather than overwhelming consumers with shifting technological novelty, Yakult has cultivated an exhaustive clinical repository evaluating this single strain’s efficacy across digestive transit, oral health, immunomodulation, stress-related physiological outcomes, and broader gut-immune benefits [110,111,112,113,114,115,116,117]. This deep strain-specificity allows the brand to deploy clear, non-technical messaging that minimizes cognitive overload while preserving scientific credibility and authority. Globally, Yakult maintains strict standard operating procedures by utilizing an identical mother strain originating from Japan across all international manufacturing hubs [109]. To navigate diverse market identity, the brand dynamically adjusts its packaging volumes to regional regulatory requirements and consumer habits, distributing 65 mL bottles across Europe, Australia, New Zealand, India, Indonesia, and Vietnam; 80 mL bottles in the Americas, Japan, the Philippines, Thailand, Malaysia, and South Korea; and 100 mL bottles in Singapore, Hong Kong, Taiwan, and China [118].

When mapped against the trust architecture proposed in Figure 1, Yakult serves as a premier example of a successful technological internalization pathway. By relying on a deeply established dairy vehicle to leverage the familiarity vector, Yakult minimized initial socio-cognitive resistance. Over decades, the brand systematically satisfied the framework’s regulatory credibility and scientific validation vectors via ongoing clinical publication [119]. This continuous alignment directly reinforced perceived safety, creating a resilient baseline of trust that lowered the psychological barriers typically associated with ingesting live bacterial cultures.

Key insight: Yakult demonstrates that long-term marketplace trust is sustained not through continuous technological complexity, but through strain-specific consistency, manageable consumer messaging, and localized structural adaptation.

### 6.2. Successful Case: Activia (Danone)—Mainstreaming Probiotics Through Evidence and Familiarity

Danone’s Activia demonstrates how complex probiotic innovations can reach mass-market scale when clinical substantiation is embedded within accessible, everyday consumption habits [120]. Activia’s core probiotic strain, *Bifidobacterium animalis* subsp. *lactis* DN-173 010 (CNCM I-2494), is backed by a robust portfolio of randomized controlled trials documenting improvements in colonic transit times and digestive comfort [121,122]. Crucially, Danone expanded its testing parameters to evaluate the complete yogurt matrix itself rather than isolated strain data [122,123,124]. This comprehensive evidence base allowed the brand to make explicit, tangible claims “regulates intestinal transit” and “helps support digestive comfort,” that directly bypass the consumer skepticism typically triggered by vague immunity narratives.

Activia’s market architecture relies on reducing novelty risk. By delivering probiotics via a culturally ubiquitous food matrix (yogurt), the brand integrates functional supplementation into existing eating routines without demanding behavioral shifts. Furthermore, the brand has demonstrated high regulatory responsiveness. Following the implementation of EFSA’s strict health and digestive-transit claims criteria under Regulation (EC) No. 1924/2006, Danone systematically realigned its European labeling, shifting toward structural front-of-pack transparency and health-professional outreach to communicate efficacy without incurring regulatory penalties [125,126].

In the framework of Figure 1, Activia successfully harmonized the familiarity and innovation axes. By translating abstract microbial activity into experienced, benefit-driven narratives regarding digestive regulatory health, the brand satisfied the framework’s scientific validation prerequisites while utilizing a traditional food matrix to shield consumers from technology neophobia, driving a positive shift across the trust continuum [127,128,129].

Key insight: Activia proves that embedding verified scientific validation within a highly familiar, culturally accepted food format overcomes consumers’ risk aversion and mainstream-scale friction.

### 6.3. Failed Case: Danone Essensis—When Positioning Outpaces Evidentiary Credibility

Danone’s Essensis “beauty yogurt” offers a stark structural contrast, illustrating how ambitious positioning can cause complete consumer rejection when claims decouple from intuitive plausibility and empirical validation. Launched across European markets as a “dermo-nutrition” breakthrough, Essensis was marketed on a “beauty from within” narrative, claiming that regular consumption could significantly mitigate cutaneous water loss and restore skin health [130]. However, the product immediately triggered profound public and consumer watchdog skepticism, with organizations like UFC-QC challenging the tangible efficacy of the intervention [131,132]. The failure of Essensis traces two distinct cross-vector mismatches within the Figure 1 architecture. First, the promised benefit was completely uncoupled from immediate bodily experience. Unlike digestive transit, which can be noticed more readily and linked to immediate bodily experience, skin hydration changes occur slowly and are mediated by complex external variables, making the causal link highly abstract for many consumers [131].

Second, a severe category disconnects existed between the product format (refrigerated yogurt) and the cosmetic beauty promise. Because the biological link between ingestible dairy and dermatological improvement was not intuitively aligned with the consumer’s naturalness heuristic, the cognitive load spiked. Lacking the transparent, mechanistic explanations required to overcome this cognitive distance, the product could not justify its premium price tier [131,132]. The perceived safety vector inverted into techno-skepticism, precipitating a total systemic breakdown and its eventual withdrawal from the market.

Key insight: Marketing narratives fail when the functional promise fundamentally clashes with the product format’s intuitive boundary, creating a trust gap that promotional spending cannot bridge.

### 6.4. Probiotic Waters and “Gut-Health Shots”: When Technology Falters in Practice

Functional probiotic waters and shelf-stable “gut-health shots” represent a rapidly growing market segment where ambitious engineering concepts frequently falter due to structural formulation limits, volatile shelf-life stability, and sensory divergence [133,134].

A central challenge is keeping the bacteria alive. Probiotics are living microorganisms that require a suitable pH balance, low oxygen, and controlled temperature to survive and remain functional. In oxygen-rich, shelf-stable liquid formats, many strains deteriorate rapidly, so viable counts may fall below label claims before the product is consumed [133]. When independent testing shows lower-than-advertised colony-forming units, consumer trust is undermined because the product fails to deliver on its core scientific promise: live microbes in sufficient numbers [133,135]. Sensory performance is another critical weak point for these water-based drinks. Empirical profiling of alternative probiotic matrices indicates that shelf-life progression frequently triggers the development of volatile off-flavors, a bad smell, unappealing sedimentation at the bottom, or visible changes over shelf life, or a separation of liquids. Consumer adherence and product acceptance are strictly dependent on sensory congruence [55]. When unpalatable deviations occur, consumers instinctively apply cognitive shortcuts, often interpreting these sensory shifts as indicators or signs of poor quality, microbial spoilage, or systemic formulation failure. This is especially problematic for products marketed as “clean,” “pure,” or “functional wellness” items because any sensory inconsistency clashes with the expected image of freshness and reliability.

Overgeneralized health claims further weaken credibility [136]. Claims such as “supports immunity,” “detoxifies the body,” or “boosts gut health” are difficult to substantiate clearly and can attract regulatory scrutiny and reinforce consumer skepticism [136]. Without clear label information about the exact strain used, the dose, and how to store the product, consumers cannot judge whether the product is scientifically plausible or merely marketing hype.

In the context of the Figure 1 framework, these new formats lack the historical familiarity of traditional options like yogurt. When consumers encounter unexpected off-flavors or visible flaws in a new type of drink, it triggers food technology neophobia (a fear of new food technologies) and breaks consumer acceptance.

Key Insight: Technical feasibility, cellular survival validation, and strict sensory control are mandatory prerequisites for market survival; branding cannot compensate for physical product degradation or unpalatable organoleptic deviations.

### 6.5. Cross-Case Insights: What Drives Trust and What Breaks It

Synthesizing these global case studies isolates a clear dichotomy between the drivers of consumer trust and the catalysts of market rejection, directly mapping onto the functional mechanics of the Figure 1 conceptual model. This structural divergence is codified in Table 3, which pairs successful trust-enhancing vectors with their corresponding trust-disrupting counterparts.

A visual summary mapping the four empirical case studies along the core trust vectors identified in Figure 1. Successful integrations (Yakult and Activia) are plotted, showing a balanced alignment where robust scientific validation and high product familiarity insulated consumer-perceived safety. Market failures or disruptions (Essensis and functional probiotic waters) are mapped to show structural friction points, demonstrating how a decoupling from consumer familiarity expectations or sensory/stability failures causes the naturalness heuristic to invert into rejection. This cross-case matrix underscores that consumer trust is not an automated consequence of advanced biotechnological innovation. Rather, it is a dynamic socio-cognitive integration process. Products succeed when complex microbial science is translated into manageable value propositions, recognizable vehicles, and transparent narratives. Conversely, when this translation breaks down or fails to account for inter-individual microbiota variability, consumer confidence erodes, moving the product from an acceptance pathway into a failure vector.

## 7. Regulatory Frameworks and Governance of Probiotics

The governance of probiotics is complex, fragmented, and highly variable across jurisdictions, and this regulatory landscape shapes how next-generation probiotic innovations are developed, marketed, and perceived. Unlike pharmaceuticals, probiotics are typically regulated as foods, dietary supplements, or novel foods depending on their intended use, formulation, and national definitions. Where frameworks are clear and transparent, they can strengthen confidence in probiotic safety and efficacy. Where they are ambiguous or inconsistent, they can foster skepticism, confusion, and susceptibility to misinformation.

### 7.1. Global Regulatory Landscape

To systematically evaluate how divergent risk-benefit paradigms manifest globally, Table 4 maps the foundational differences between the four primary regulatory jurisdictions governing live microbial cultures. This comparative breakdown highlights a fundamental philosophical schism in global food policy. On one side, the European Union operates under a precautionary, centralized model where safety is pre-appraised via taxonomic groups (the QPS framework), but functional marketplace messaging is heavily restricted under an implied-claim ban. On the other side, the United States employs a decentralized, post-market notification system (GRAS) that allows agile, structure-function health claims but shifts the burden of strain accountability onto corporate liability. Navigating between these blocks are nations like Canada and Japan, which utilize rigorous, pre-market clinical vetting but provide clear, structured legal pathways, such as Japan’s Foods for Specified Health Uses (FOSHU) system, for brands to explicitly market strain-specific clinical efficacy directly to consumers.

- European Union (EU)

In the European Union, probiotics fall under overlapping regulatory frameworks depending on composition and intended use. The European Food Safety Authority (EFSA) applies the Qualified Presumption of Safety (QPS) approach to microbial safety assessment, which provides a streamlined, generic pre-evaluation of safety at the taxonomic unit level based on taxonomic identity, the body of relevant knowledge, and the absence of pathogenic traits [137,138]. While the QPS system establishes baseline safety for approved species, the assessment of functional efficacy remains strictly bound to the strain level. This reflects a global scientific consensus that probiotic health effects are strain-specific rather than species-wide. This scientific consensus is that probiotic effects are strain-specific rather than species-wide [2].

However, EFSA maintains a highly restrictive stance on health claims. Under Regulation (EC) No. 1924/2006 on nutrition and health claims, probiotic-specific health claims have not been authorized at the EU level because of the systemic difficulty of demonstrating universally applicable, clear cause-and-effect relationships for specific strains in healthy populations [139].

As a result, the term “probiotic” itself is officially interpreted as an implicit health claim, and its inclusion on commercial packaging is restricted or discouraged across several Member States. While this stringent approach promotes scientific rigor and reduces the risk of overstatement and consumer deception, it also creates substantial barriers to commercial innovation and limits the ability of brands to communicate functional benefits directly to the public. Conversely, this regulatory caution can also reinforce long-term consumer trust in the institutional system, as market-approved, EFSA-aligned products are perceived as highly vetted and evidence-based.

- United States (US)

In the United States, probiotics are usually regulated as foods or dietary supplements unless they are intended to treat, mitigate, or cure a disease, in which case they fall strictly under drug regulation pathways. The vast majority of commercial probiotic strains enter the market through Generally Recognized as Safe (GRAS) determinations, either via FDA notification or through an independent panel of expert review without direct agency vetting. Alternatively, if a microbial strain was not marketed as a dietary ingredient in the US before 15 October 1994, it requires a New Dietary Ingredient Notification (NDIN) under Section 413(a)(2) of the Federal Food, Drug, and Cosmetic Act [140,141]. This process mandates that manufacturers submit extensive safety and identity data to the FDA at least 75 days before introducing the ingredient into interstate commerce.

Under the Dietary Supplement Health and Education Act (DSHEA), manufacturers are legally permitted to use structure–function claims (e.g., “supports digestive health”), but they are explicitly prohibited from making disease-treatment or prevention claims without undergoing the rigorous pre-market investigational drug approval process [142].

This comparatively flexible framework supports market diversity and innovation, including a wide range of strains, formats, and delivery systems. However, it also places greater responsibility on manufacturers to ensure product safety, quality, and truthful communication. The relative permissiveness of the US system can encourage innovation, but it can also contribute to inconsistent product quality and variable claim standards, which may undermine consumer confidence when independent testing reveals discrepancies between advertised and actual characteristics.

- Canada

Canada regulates probiotics as Natural Health Products (NHPs), requiring pre-market approval, safety assessment, and strain-specific efficacy data. This model sits between the EU’s claim-focused restrictions and the US’s more flexible, largely post-market approach. By combining scientific substantiation with formal pre-market review, Canada’s system aims to balance innovation with consumer protection. Probiotic NHPs must demonstrate safety and a plausible benefit, and labels are closely scrutinized for allowable claims. This hybrid approach tends to foster relatively high institutional trust, since consumers may perceive that products have undergone formal scientific review before reaching the market.

- Asia-Pacific

Japan’s Foods for Specified Health Uses (FOSHU) system is among the most established and rigorous probiotic regulatory frameworks. FOSHU products undergo pre-market evaluation of safety and efficacy, including strain-specific human studies, and are authorized only when a clear cause-and-effect relationship has been demonstrated. South Korea and China employ similar pre-market approval systems with strong institutional oversight and strain-specific evidence requirements. These systems support comparatively high consumer trust in regulatory authorities because probiotic products are seen as subject to stringent scientific scrutiny before commercialization.

The EFSA QPS system and the FDA GRAS paradigm both emphasize strain-level characterization, aligning with the broader scientific consensus that probiotic effects cannot be reliably extrapolated across species or genera. International standards such as ISO 19344:2015 [143] provide additional technical guidance for the quantification of probiotic strains in dairy products, although adoption remains voluntary and implementation varies by country. Where enforcement is weak, such as in markets where incomplete strain identification or insufficient viability data persist, consumer trust can be undermined, especially when independent testing reveals discrepancies between label claims and measured outcomes.

### 7.2. Safety Assessment and Quality Standards

Despite regional differences, several core principles underpin probiotic safety assessment across jurisdictions. Accurate taxonomic identification, typically supported by genomic methods, is now regarded as a baseline requirement because probiotic properties are strain-specific. Screening for antibiotic resistance genes, evaluation of virulence factors, toxicological and metabolic profiling, and demonstration of strain-specific safety are also widely emphasized. Regulatory frameworks increasingly require verification of viable cell counts at the end of shelf life, ensuring that products deliver the microbial load they advertise.

This aligns directly with the official EFSA Activities Reporting and Roadmap for 2025 [144], which prioritizes harmonized microbiological hazard metrics across member states, and the EFSA Programming Document 2025–2027 [145], which outlines the systematic integration of updated scientific data architectures to independently communicate food chain risks. This updated framework underscores that microbiome-informed safety assessment requires harmonized methods and translational models, reinforcing the role of regulatory science in trust-centered innovation [126]. In this context, robust safety assessment and quality control do not only ensure scientific legitimacy; they also function as trust-building mechanisms by signaling that products have undergone rigorous strain-specific evaluation.

### 7.3. Governance Challenges: Claims, Viability, and Transparency

- Health claims

Regulation of health claims remains one of the most contested areas in probiotic governance. The EU’s stringent requirements limit approved claims, effectively constraining marketing language but reinforcing expectations of scientific rigor. In contrast, more permissive systems in the US and parts of Asia allow broader structure–function or benefit-oriented claims, which can support innovation and product differentiation but also create opportunities for exaggerated or misleading statements. This global fragmentation highlights the distinct regulatory hurdles faced when classifying probiotic products along the spectrum of conventional foods, dietary supplements, or live biotherapeutic products (LBPs) [11]. The resulting inconsistencies can confuse consumers, particularly when similar products carry very different claims in different markets. Furthermore, these international structural variations continue to complicate global taxonomy standards and the official designation of strain-level properties, particularly as regulatory frameworks struggle to keep pace with the differentiation between traditional strains and newly isolated next-generation probiotics [146].

- Viability and label accuracy

Ensuring that products contain the labeled number of viable microorganisms at the end of shelf life remains a persistent technical and regulatory challenge. Discrepancies identified through independent testing can erode consumer confidence, especially in an environment where live microbial cells are central to the product’s promised benefit. This underscores the ongoing governance challenge of ensuring uniform viability metrics and standardized manufacturing practices across cross-border supply chains [11]. In response, regulatory attention is increasingly shifting toward strain-specific labeling, guaranteed viable counts through expiry, clear storage instructions, and verification of shelf-life stability. These measures aim to close the gap between marketing claims and real-world performance.

- Transparency and traceability

Modern governance frameworks increasingly emphasize transparency in strain identity and origin, production and fermentation processes, safety assessments, and quality control procedures. Digital traceability tools, including blockchain-enabled systems and batch-level QR-linked ingredient pathways, are emerging as governance innovations that enhance accountability and allow consumers to verify product claims more directly. In a digital environment where both trust and suspicion spread quickly, these mechanisms can help signal that probiotic products are scientifically sound as well as traceable and verifiable.

### 7.4. Ethical and Social Dimensions of Probiotic Governance

Beyond technical regulation, probiotic governance must also address broader ethical and societal concerns. Equity and access are central issues because governance frameworks should prevent probiotic benefits from being limited to high-income populations. Cultural appropriateness is also important since regulatory systems should respect traditional fermented foods and local microbial knowledge systems rather than imposing a single industrial model across diverse food cultures. These equity concerns interface directly with public health stewardship; the intentional manipulation of shared or community microbiomes necessitates long-term safety monitoring and ethical oversight to protect vulnerable populations [147].

Corporate responsibility is equally important. Preventing exaggerated claims and enforcing evidence-based marketing are essential to maintaining long-term public trust. Ethical governance models must actively address public health implications, shifting the industry toward a paradigm of responsible “microbial stewardship” rather than purely commercial exploitation [147]. In the case of precision-fermented ingredients and engineered microbial strains, data transparency becomes particularly salient, as consumers and civil-society actors increasingly seek clear information about genetic modifications, production conditions, and environmental impacts. Integrating these dimensions into governance frameworks is not a side issue; it is a core component of trust-centered regulation.

### 7.5. Toward Harmonization and Trust-Centered Governance

There is growing international interest in harmonizing probiotic regulations to reduce fragmentation and improve clarity for both consumers and industry. Key priorities include standardizing strain-specific labeling practices, aligning evidence requirements for health claims, establishing global quality and safety benchmarks, and expanding digital traceability systems. Harmonization efforts could also promote more consistent communication norms, reducing the patchwork of claim standards and label formats that currently confuse consumers and complicate cross-border trade. This is particularly critical because regulatory fragmentation and conflicting health claims directly degrade public trust in scientific authority, necessitating unified cross-border policies to protect consumers and improve public health messaging [148]. Furthermore, establishing harmonized evaluation frameworks across academia, industry, and regulatory authorities is essential for balancing industrial viability with rigorous human safety and efficacy standards as next-generation formulations scale [149].

A trust-centered governance model, combining scientific rigor, regulatory transparency, and active consumer engagement, offers the most promising pathway for supporting innovation while maintaining public confidence. In this model, regulatory systems are not merely control mechanisms but active contributors to trust formation: they signal safety, endorse evidence-based claims, and create the conditions under which technological innovation can proceed in ways that are perceived as legitimate, transparent, and socially responsible. In the evolving landscape of next-generation probiotics, such governance frameworks will be essential for reconciling scientific advancement with the slower, socially embedded process of trust building.

**Figure 6:**
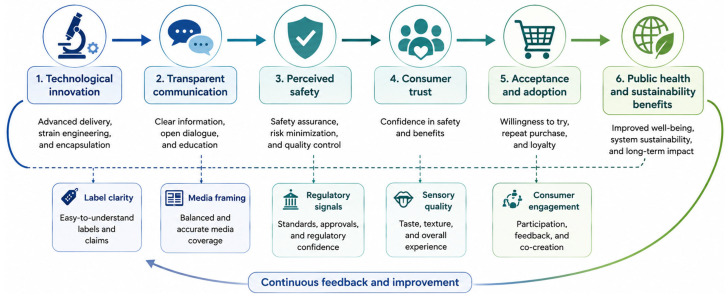
Pathway from probiotic innovation to consumer acceptance.

Figure 6 presents the pathway through which probiotic innovations move from technological development to consumer acceptance. Transparent communication, credible regulatory signals, and positive sensory expectations mediate the relationship between innovation and trust, while consumer engagement helps convert perceived safety into adoption and broader public health benefits.

This pathway highlights that market success depends on more than efficacy alone; it requires a coordinated trust-building environment across product design, labeling, regulation, and public communication. By demonstrating how technological development, transparent communication, and regulatory credibility contribute directly to perceived safety, Figure 5 maps out the sequential stages required for consumer trust and eventual adoption of next-generation probiotic products. However, it is critical to recognize the structural limitations of the ‘trust through transparency’ paradigm. Transparency is not an absolute remedy for consumer skepticism; its efficacy is strictly bound by the nature of the consumer’s objection. While information disclosure can effectively mitigate food neophobia or clarify misunderstood scientific processes, it is largely ineffective when confronting deeply held moral, ethical, or ideological values. When a consumer operates under an absolute moral rejection of a technology, such as specific genetic modifications or synthetic biology interventions, increased transparency can induce an informational backfire effect [150]. By fully disclosing technical frameworks, communication strategies may inadvertently highlight the exact attributes that trigger ideological resistance, thereby reinforcing market rejection rather than fostering acceptance [151]. Consequently, the trust-centered governance model proposed here must be understood not as a mechanism to override deeply held human values but as a framework optimized for contexts where risk perception is driven by informational asymmetry rather than immutable ethical boundaries.

## 8. Discussion

The rapid evolution of probiotic technologies, including micro- and nanoencapsulation, precision fermentation, engineered strains, and postbiotic formulations, has created new opportunities to improve the stability, efficacy, and personalization of probiotic-enriched functional foods. However, the preceding sections show that technological advancement alone does not guarantee market success. Consumer trust remains the central determinant of acceptance, making it essential to align innovation with perception, communication, and governance.

Across the literature, trust emerges as a multidimensional outcome shaped by psychological factors, sensory expectations, socio-cultural norms, and prior experience. Health consciousness, neophobia, and risk–benefit appraisal influence how consumers interpret the promise of probiotic products, while labeling, digital traceability, media narratives, and regulatory signals act as external filters that shape perceptions of safety, naturalness, and credibility. Trust is therefore not simply a response to scientific evidence but the result of a broader evaluative ecosystem.

Sensory quality is equally central. Even technically advanced formulations are unlikely to succeed if they compromise taste, texture, or appearance. Products that preserve sensory familiarity allow consumers to adopt functional benefits without feeling that enjoyment has been sacrificed. In probiotic foods, sensory congruence is not an accessory feature but a core condition of trust and repeated consumption.

Transparency and communication are also decisive. Clear labeling, accessible explanations of microbial processes, and digital traceability tools reduce uncertainty and help consumers make informed choices. By contrast, overly technical or opaque communication increases cognitive burden and can weaken confidence even when the underlying science is sound.

The media ecosystem adds another layer of complexity. Social media can amplify misinformation, fear-based narratives, and exaggerated claims, especially around engineered microbes and precision-fermented ingredients. Yet the same platforms can also support trust through educational content, expert engagement, and transparent storytelling. Media, therefore, does not merely reflect public opinion; it actively shapes the terms on which probiotic innovations are interpreted.

Regulatory frameworks function as trust signals rather than neutral backdrops. The fragmented global landscape, spanning the EU’s restrictive claim-based model (e.g., the European Food Safety Authority’s (EFSA)), the U.S. structure–function framework (e.g., the U.S. Food and Drug Administration’s (FDA)), and pre-market approval systems in parts of Asia, creates different conditions for innovation and consumer confidence. Greater harmonization around strain-specific labeling, viability requirements, and claim substantiation would reduce confusion and strengthen legitimacy. Governance must also address ethical dimensions, including transparency, equity of access, responsible marketing, and respect for traditional fermented foods.

The case studies reinforce these patterns. Successful products combine clear and relatable health benefits, strong sensory performance, transparent communication, robust scientific substantiation, and regulatory alignment. Failed products, by contrast, tend to rely on vague or exaggerated claims, sensory mismatch, insufficient transparency, or technological complexity that does not translate into clear consumer value. As demonstrated in the preceding case studies, the sustained market success of traditional functional products like Yakult and Activia stems from their alignment with the proposed framework, utilizing long-term scientific validation and matrix familiarity to reinforce perceived safety. Conversely, the market rejection of Essensis and various probiotic waters illustrates how deficiencies in sensory congruence (e.g., off-flavors interpreted as poor quality) or an inability to overcome the naturalness heuristic can abruptly disrupt consumer acceptance.

Trust, in other words, is built when complex microbial science is translated into familiar, credible, and meaningful everyday benefits. Ultimately, these dynamics support an integrated model that pairs downstream consumer heuristics with upstream microbial design. As probiotic technologies continue to advance through engineered strains, postbiotic formulations, and precision-fermented bioactives, the central challenge will be to ensure that innovation remains aligned with societal expectations and lived consumer experience.

## 9. Knowledge Gaps and Future Research Priorities

Although the literature on probiotics, functional foods, and consumer trust continues to expand, several important gaps remain in the current evidence base. First, research on consumer responses to next-generation probiotic platforms, including engineered strains, precision-fermented ingredients, and advanced delivery systems, is still limited and uneven across product categories [152,153]. Prior reviews have tended to focus either on technological performance or on general consumer acceptance of precision fermentation and functional foods, leaving less attention to how consumers evaluate highly engineered probiotic innovations in relation to safety, authenticity, and perceived naturalness [36,154,155].

Second, relatively few studies examine how trust develops over time as consumers gain exposure, experience, and familiarity with regulatory processes. Much of the existing literature captures attitudes at a single point in time, which makes it difficult to understand whether trust is stable, accumulative, or contingent on repeated interactions with products, brands, and institutions [9]. Longitudinal evidence would be especially valuable for clarifying whether initial skepticism toward novel probiotic technologies diminishes as products become more familiar or as consumers encounter clearer scientific and regulatory information [21].

Third, cross-cultural evidence remains insufficient, particularly regarding how different populations interpret naturalness, technological complexity, and institutional credibility [156]. Consumer perceptions of microbial technologies are likely to vary across cultural settings, food traditions, and regulatory environments, yet many studies are concentrated in a small number of countries or rely on relatively homogeneous samples [157]. More comparative research is needed to determine how cultural values, food norms, and trust in institutions shape acceptance of next-generation probiotic products [157].

Fourth, additional work is needed on how labeling, digital traceability, and media narratives shape perceived safety in real-world settings. Although transparency is widely discussed as a trust-building strategy, less is known about which forms of communication are most effective, which information consumers actually use, and how online environments influence interpretations of risk and benefit [73]. This is particularly important in digital contexts, where algorithmic exposure, influencer messaging, and platform-specific narratives may amplify uncertainty or reinforce confidence in ways that differ from conventional food communication channels. Future research should examine how regulatory clarity, consumer education, and participatory communication can be aligned to support responsible innovation. Research that integrates consumer science, microbiology, and governance could help identify communication and policy approaches that improve understanding, reduce perceived risk, and support informed acceptance of emerging probiotic technologies.

## 10. Conclusions

The interplay between technological innovation and consumer trust will define the future of probiotic-enriched functional foods. As the sector moves toward increasingly sophisticated platforms—micro- and nanoencapsulation, biopolymer-based microbial delivery systems, engineered strains, and precision-fermented postbiotics—the potential for enhanced stability, targeted efficacy, and personalized health benefits is substantial. Yet without robust consumer trust, even the most scientifically sound innovations risk limited uptake, market failure, or public resistance.

This review shows that trust is not built on scientific evidence alone. Psychological factors such as health consciousness, neophobia, and perceived naturalness; sensory experiences of taste, texture, and appearance; socio-cultural values related to familiarity and tradition; and economic considerations such as price–value trade-offs all shape how consumers evaluate probiotic technologies. These dimensions must be addressed alongside structural conditions such as transparent labeling, traceable supply chains, and clear communication of microbial production processes.

The broader media ecosystem further complicates this landscape. Social media platforms can amplify misinformation, fear-based narratives, and exaggerated claims, yet they also offer powerful opportunities for transparent, engaging, and evidence-based communication. Brands, regulators, and scientists must therefore engage proactively, not only to counter misinformation but also to explain how probiotic ingredients are developed, why they are considered safe, and what scientific evidence underpins their benefits.

To succeed, innovation in probiotics must be embedded in trust-oriented governance. Key priorities include adopting layered, consumer-friendly communication strategies that make microbial science accessible; investing in digital traceability and transparent disclosure of strain identity, production methods, and safety assessments; engaging authentically with digital media and leveraging credible expert voices; and involving consumers through co-creation, feedback mechanisms, and participatory risk communication.

By aligning technological progress with transparency, ethical engagement, and strong sensory performance, the probiotic sector can build durable consumer trust. In doing so, it can unlock the full potential of next-generation microbial innovations to contribute to public health, sustainability, and long-term well-being.

## Figures and Tables

**Table 1:** Conceptual framework of consumer trust and behavioral acceptance.

Dimension	Tier 1: Traditional Probiotics (e.g., Lactobacillus)	Tier 2: Postbiotics (Inanimate Microorganisms/Metabolites)	Tier 3: Engineered Live Biotherapeutics (LBPs/GMOs)
Primary Trust Anchor	Familiarity and History of Safe Use	Scientific Competence and Efficacy Claims	Institutional Oversight and Biosecurity
The “Naturalness” Hurdle	Low; perceived as an extension of traditional fermentation.	Moderate; requires overcoming the “inanimate/dead” efficacy paradox.	High; triggers deep-seated “Frankenfood” and synthetic manipulation biases.
Cognitive Dissonance Trigger	None; aligns with standard wellness heuristics.	The Animation Gap: Consumers struggle to trust that a “dead” element provides active health.	The Contamination Anxiety: Consumers fear genetic horizontal gene transfer or environmental escape.
Regulatory Proxy Trust	High reliance on self-affirmed safety or GRAS status.	Requires clear labeling clarity regarding non-viability without sounding “spoiled.”	Absolute dependence on strict clinical trial transparency and GMO authorization

**Table 2:** Methodological literature search and thematic curation matrix (2020–2025).

Thematic Category	Core Conceptual Scope	Representative Target Literature from Pool
Tier 1: Next-Gen Probiotics and Matrices	Evolution of functional vehicles, plant-based and dairy innovations, candidate identification, and viability baselines.	Lalowski & Zielińska (2024) [43]; Jan et al. (2024) [4]; Uhegwu & Anumudu (2025) [44]; Grujović et al. (2025) [45].
Tier 2: Advanced Delivery and Physical Solutions	Microencapsulation, nanoencapsulation, structured hydrogels, and 3D printing food delivery systems.	de Oliveira Filho et al. (2025) [46]; Sethunga et al. (2025) [47]; Szpicer et al. (2025) [48]; Multisona et al. (2025) [49].
Tier 3: Precision Fermentation and Genetic Tools	Synthetic biology tools, precision proteins, AI-driven hyper-personalized diets, and molecular optimization.	Marcellin et al. (2024) [37]; de Almeida et al. (2024) [50]; Ali et al. (2025) [51]; Priyadharshini et al. (2025) [52]; Ajayeoba & Ijabadeniyi (2025) [53]; Peiris et al. (2025) [54]
Socio-Cognitive Barriers and Consumer Insights	Tech-neophobia, sensory congruence, contrasting public perceptions, and the “naturalness heuristic.”	Bogueva & Danova (2024) [36]; Tonacci & Gorini (2025) [55]; Zhao et al. (2025) [56]; Alalwan (2025) [57].
Regulatory Governance and Biosecurity	Novel foods, farm-to-fork initiatives, institutional policy foresight, and legal baselines.	Lowe, Minssen & Skentelbery (2024) [58]; Nwakoby et al. (2025) [59]; Mihai et al. (2025) [60].

**Table 3:** Comparative success and failure matrix of historical case studies.

Trust-Enhancing Vectors (Successful Launches)	Trust-Disrupting Vectors (Failed Launches)
Clear benefit realization: Easily perceived bodily outcomes (e.g., digestive regulatory tracking).	Abstract functional distance: Non-verifiable or delayed effects (e.g., nutricosmetic skin claims).
Matrix familiarity: Cultural integration via traditional vehicles (e.g., fermented dairy formats).	Category: Mismatched product vehicles forcing high cognitive processing.
Strain-specific transparency: Unwavering, long-term commitment to single, verified biological agents.	Opaque characterization: Unvalidated strain identities or generic, unquantified claims.
Sensory congruence: Predictable organoleptic profiles that reinforce perceived freshness and safety.	Organoleptic fluctuation: Phase separation or off-flavors interpreted as spoilage.
Proactive compliance: Adaptation to rigorous institutional validation structures (e.g., EFSA, FDA, and NDIN).	Overgeneralized positioning: Vague marketing language that collapses under regulatory or legal scrutiny.

**Table 4:** Comparative matrix of global probiotic regulatory frameworks.

Jurisdiction	Primary Regulatory Classification	Pre-Market Approval Required?	Health/Functional Claim Allowances	Strain-Specific Requirements
European Union (EU)	Food/Dietary Supplement/Novel Food	Yes (if classified as Novel Food)	Highly restricted; “probiotic” is treated as an implied health claim and generally rejected.	Strict; mandatory strain characterization under EFSA QPS guidelines.
United States (US)	Dietary Supplement/Food Ingredient	No (unless making drug claims; utilizes GRAS notification)	Structure-function claims allowed without pre-market vetting; disease claims prohibited.	Moderate; self-affirmed but strain specificity expected for corporate liability.
Canada	Natural Health Product (NHP)	Yes (requires product license)	Permissible conditional on clinical evidence portfolios.	High; strain-specific characterization required for NHP approval.
Japan	Foods for Specified Health Uses (FOSHU)	Yes (stringent scientific vetting)	Specific, approved functional health claims permitted upon proof of clinical efficacy.	Very High; trial data must correspond exactly to the commercialized strain.

## Data Availability

The original contributions presented in this study are included in the article. Further inquiries can be directed to the corresponding author.
